# Intimate partner violence during COVID-19 lockdown in Norway: the increase of police reports

**DOI:** 10.1186/s12889-021-12408-x

**Published:** 2021-12-16

**Authors:** Merete Berg Nesset, Camilla Buch Gudde, Gro Elisabet Mentzoni, Tom Palmstierna

**Affiliations:** 1 Forensic Department and Research Centre Brøset, St. Olav’s Hospital, Trondheim University Hospital, PO 1803 Lade, N-7440, Trondheim, Norway; 2 Trøndelag Police District, Crime Prevention Department, The Children’s House, Trondheim, Norway; 3 Faculty of Medicine and Health Sciences, Department of Mental Health, Norwegian University of Science and Technology (NTNU), Trondheim, Norway

**Keywords:** COVID-19, corona virus, lockdown, intimate partner violence, domestic violence, public health, prospective cohort study, violence risk assessment

## Abstract

**Background:**

In March 2020, the Norwegian government announced a COVID-19 lockdown in order to reduce the spread of the coronavirus. In Norway, lockdown measures included restricting people’s ability to leave their home and the closing of social institutions, thus reducing the capacity for victims of intimate partner violence to alert someone outside of their home about violent incidents that occurred during lockdown. At the same time, the restrictive measures forced the victim and the perpetrator to stay together for prolonged periods within the home, and reduced the possibility for them to escape or leave the perpetrator. The aim of this study was to investigate how the frequency and character of intimate partner violence reported to the police changed during the period of lockdown in Norway.

**Methods:**

All cases of intimate partner violence registered in police files before the pandemic (from January 2016-February 2020) and during lockdown in Norway (March-December 2020) were included in the study, representing a total of 974 cases. Differences in the number and severity of cases were calculated using χ2-tests and Wilcoxon’s rank sum test. Differences in the characteristics of the reported violence was assessed with the Brief Spousal Assault form for the Evaluation of Risk (B-SAFER) and tested with Fischer’s exact test with Bonferroni correction. Standardised Morbidity Rate (SMR) statistics were used to analyse the proportion of immigrants as compared to the general population.

**Results:**

Reported intimate partner violence increased by 54% during the lockdown period in Norway. Between March-December 2020, the police assessed the cases as being at higher risk of imminent and severe violence. Our findings indicated an overrepresentation of immigrant perpetrators before and during lockdown (SMR = 1.814, 95% CI = 1.792–1.836 before, and SMR = 1.807, 95% CI = 1.742–1.872 during lockdown). Notably, while victims with an immigrant background were overrepresented before lockdown, we found significantly lower proportion of immigrant IPV victims during the lockdown period (SMR = 1.070, 95% CI = 1.052–1.087 before, and SMR = 0.835, CI 95% CI = 0.787-0.883 during lockdown). Also, there were significantly more female perpetrators and male victims reported to the police during the lockdown period. A higher proportion of the victims were assessed as having unsafe living conditions and personal problems during lockdown. Finally, during the lockdown period in Norway, a higher proportion of perpetrators had a history of intimate relationship problems.

**Conclusions:**

Intimate partner violence increased dramatically during the COVID-19 lockdown. A range of options for victims to escape from their perpetrators, particularly during times of crisis, should be developed in line with good practice, and with a special focus on the most vulnerable victims.

## Background

Intimate partner violence (IPV) was a worldwide health problem even before the COVID-19 pandemic, with a lifetime occurrence of physical and/or sexual violence by an intimate partner estimated to be 30% for women globally [1]. Intimate partner violence and violence against children have been described as a ‘hidden pandemic’ within the COVID-19 pandemic [2]. Furthermore, research has shown that during other crisis situations intimate partner violence has also increased, for example, during natural disasters [3]. The COVID-19 pandemic has led to rapid changes in peoples’ daily routines, stay-home policies and lockdown of social institutions, such as schools and health centres. Furthermore, reduced capacity and more remote access to public institutions, including the police, health- and social services has led to a general concern about the situation for victims of intimate partner violence during the pandemic [4–7].

Although there is little scientific knowledge about the consequences of the COVID-19 pandemic on family violence, there are indications that social distancing and lockdowns of societies have increased family conflicts [8, 9]. A recent systematic review and meta-analysis of 18 studies from countries around the world excluding Norway, found that intimate partner violence had increased after COVID-19 lockdowns (mean effect size 0.66, CI = 0.08–1.24) [8]. Furthermore, the pandemic has caused increased stress within the home, such as increased poverty due to loss of employment, which in turn has been found to be associated with intimate partner violence [10]. Other known risk factors for intimate partner violence include lack of formal and informal support from health- and social services, friends and family, mental health problems and drug use [11, 12]. Accordingly, when social welfare institutions lock down, it is particularly important for police and health care services to be aware of, and available to victims of violence in a crisis like the COVID-19 pandemic.

Intimate partner violence includes physical violence, sexual violence, stalking and psychological aggression [13]. Exposure to violence from an intimate partner has been shown to have a long-term negative impact on the victims’ psychological and physical well-being [14–16], and to represent significant economic costs to society [17, 18]. Both men and women can be either perpetrators or victims of intimate partner violence, or they can be both. Previous research on intimate partner homicide has indicated that mutual violence between partners was common among cases that ended with homicide, with female partners being the victim of homicide in the majority of cases [19]. A meta-analysis examining the association between depression, anxiety, post-traumatic stress disorder (PTSD), antisocial personality disorder, borderline personality disorder, and physical intimate partner violence perpetration and victimisation among men and women, found a strong link between all of the studied mental health disorders and violence perpetration and victimisation for men and women [11].

Identifying intimate partner violence requires robust assessment procedures within police, health- and social services. The assessment of risk for intimate partner violence may be defined as *the process of gathering information about people to make decisions regarding their risk of perpetrating intimate partner violence* [20]. The aim of risk assessments for violence is to provide a sound basis for implementing effective protective measures and management plans to hinder future incidences of violence [21]. The Brief Spousal Assault Form for the Evaluation of Risk (B-SAFER) is such a structured professional judgement device designed for use in police and other criminal justice settings [22].

Importantly, police officers are often the first line responders to intimate partner violence, and evidence suggests that women exposed to potentially lethal violence are more likely to seek help from the police or health services [19]. Although previous research has examined characteristics of intimate partner violence, there is a need for much more research on the consequences of national lockdowns due to the COVID-19 pandemic in the number and severity of intimate partner violence cases. In addition, little is known about specific risk and vulnerability factors among perpetrators and victims associated with the pandemic lockdown situation, as assessed by the police. Furthermore, the knowledge about how the pandemic has influenced immigrants as victims and perpetrators of intimate partner violence is still limited.

The aim of this study was to investigate changes in character and occurrence of intimate partner violence (IPV) reported to the police during lockdown as compared to IPV incidents reported prior to the COVID-19 lockdown in Norway. Our pre-defined primary outcome was the number and severity of IPV cases before and during lockdown. The pre-defined secondary outcomes were differences in the characteristics of specific risk and vulnerability factors, and immigration status among perpetrators and victims before and during lockdown.

## Methods

### Design

All the data collected in this study relied upon all police reports within a defined area, Trøndelag County, Norway, including structured risk assessments conducted by specially assigned police officers. To meet the aim of the study, we compared characteristics of cases reported before the COVID-19 related lockdown in Norway in March 2020 with the same characteristics during March to December 2020. The current study is part of a prospective cohort file study of police risk assessments of all reported intimate partner violence cases in Trøndelag County, Norway since 2015. In this paper, we report findings from the full years of 2016–2020.

### Setting and Sample

On a national level, the Norwegian police force has assigned police officers working with violence in the family. These police officers are responsible for performing risk assessments and suggesting interventions, using the B-SAFER risk assessment tool to guide decisions in this process. The structured professional judgement approach encourages professionals to use their own experience when working with intimate partner violence, combined with a focus on empirically supported risk factors when assessing risk for violence and deciding on relevant interventions [20, 22].

Our study was carried out within Trøndelag Police District in Norway. Population data for the Trøndelag region was retrieved from Statistisk Sentralbyrå (Statistics Norway, the official source of population data in Norway). Trøndelag County had a mean population during 2016–2020 of 461 653 inhabitants. The mean proportion of immigrants was 10 per cent. During the period of 1^st^ January 2016 until 21^st^ December 2020, 974 IPV index cases with unique perpetrators were collected, representing all unique cases reported to the police during this period. Table 1 presents the sample characteristics.

**Table 1:** Baseline characteristics for the sample. Values presented as mean and standard deviation (SD) or proportion (%)

	**Victims (*N *= 974)**	**Perpetrators (*N* = 974)**
**Age,** mean, (SD), range	36.6 (11.5) 17–81	39.4 (12.1) 18–85
**Sex,** N (%)		
Male	57 (5.9)	951 (93.9)
Female	917 (94.1)	59 (6.1)
**Origin of birth,** N (%)		
Born in Norway	818 (84.0)	684 (70.2)
Born outside Norway	156 (16.0)	290 (29.8)

### Material and procedure

The police in Trøndelag Police Department performed B-SAFER assessments on all cases of IPV reported to the police. The data in the present study was collected and anonymized by one police employee. The anonymized ratings were collected by the researchers together with an excel-file containing sociodemographic variables, including status such as immigrant.

A Norwegian translation of the original Swedish version of the B-SAFER was used in the study [23]. As noted earlier, B-SAFER is a research-based risk assessment tool for assessing and managing the risk of future partner violence [22]. It is a 15-item risk-assessment tool that integrates both static and dynamic factors in relation to an IPV perpetrator’s risk for future violence towards an identified partner. The B-SAFER items are divided into three domains: (i) Perpetrator risk factors, (ii) the perpetrator’s psychosocial adjustment, and (iii) victim vulnerability factors (see Table 2).

**Table 2:** The Brief Spousal Assault Form for the Evaluation of Risk (B-SAFER)

**Perpetrator risk factors**	**Psychosocial adjustment**	**Victim vulnerability factors**
Violent acts	General criminality	Inconsistent behavior/attitude
Violent threats or thoughts	Intimate relationship problems	Extreme fear
Escalation	Employment problems	Inadequate access to resources
Violation of court orders	Substance use problems	Unsafe living situation
Violent attitudes	Mental health problems	Personal problems

Each factor is assessed as either *present*, *partly present*, *not present* or as ‘impossible to assess’ due to *lack of information*. The risk factors are considered to assess both the current situation (i.e. the last four weeks) and the past (prior to the last four weeks).

Finally, all the information, together with the police’s professional judgement, is summarized within two risk ratings: One for acute/imminent violence and one for severe/fatal violence. The estimated risk ratings are assessed on a three-level risk scale: Low, medium or high.

### Analyses/Statistics

Analysis of the differences in the number of IPV-cases before and during COVID-19 related lockdown in Norway reported to the police were calculated with χ2-analysis. Differences between the two periods regarding acute/imminent violence and severe/fatal violence were analysed with Wilcoxon rank sum test giving the score “1” for “Low risk”, “2” for “Medium risk” and “3″ for High risk”.

Differences in perpetrator risk factors, psychosocial adjustment and victim vulnerability factors were compared across the two time periods. All items of the B-SAFER were used for this purpose, with the exception of the items relating to ‘previous victim vulnerability’ which we lacked information about during the first years of the larger study. To exclude ‘previous victim vulnerability’ was in line with recommendations from a previous study carried out by Belfrage et al. in 2008 [24]. In accordance with our previous study on police officers use of the B-SAFER [25], item scores of ‘present’ or ‘partly present’ were judged as the risk factor being present. The scoring of “not present” was judged as the risk factor not being present. Using the B-SAFER in this way results in 25 items to analyse. The findings were calculated using Fischer’s exact test when comparing proportion of present/non-present risk factors before and during lockdown. Due to the large number of tests in this analysis, Bonferroni correction was used for accepting a significance level of 5%, i.e. only tests with *p* < 0.002 were accepted as significant.

Over- or underrepresentation of immigrants as perpetrators and victims as compared to the general population in the Trøndelag County was calculated as the quotient between observed numbers of immigrants divided by the age and sex adjusted expected number of immigrants in line with Standardised Morbidity Rate (SMR) statistics. Expected numbers of immigrants was calculated yearly since the proportion of immigrants in the general population increased from 9.1–10.8% between 2016 and 2020. Calculations were performed for the whole period, but also separately for the period before lockdown (January 2016-February 2020) and during lockdown (March 2020-December 2020).

## Results

### Number and severity of IPV cases before and during lockdown

We found a significant increase in intimate partner violence reported to the police during the lockdown period. In the study period before lockdown, i.e. between January 2016 and February 2020, 745 cases of IPV were reported, representing 14.9 cases per month (50 months in total). During the lockdown period studied, March-December 2020, 229 cases of IPV were reported, which constitutes 22.9 cases per month (10 months in total). This is an increase of 54% and statistically significant in χ2 testing with χ2 = 32.9, df = 1,* p* < 0.00001.

The police assessments of risk for acute/imminent violence was significantly higher during the lockdown period. There were 744 assessments (one missing) before lockdown that had a mean score of 1.59 and there were 229 assessments during lockdown that had a mean score of 1.67. This difference is statistically significant at the 5% level with Wilcoxon rank sum test, *p* = 0.0499.

Further, there was a trend towards a higher risk for severe reoffending among the cases during lockdown, although the difference was not statistically significant (Wilcoxon rank sum test, *p* = 0.054); The 745 assessments before lockdown had a mean score of 1.49 and the 229 assessments during lockdown had a mean score of 1.57.

### Differences in perpetrator’s risk factors and victim’s vulnerability factors before and during lockdown

As presented in Table 3 below, the perpetrator risk factors were virtually the same before and during lockdown. Current and past violent acts were very similar. What differed was that in the lockdown group, there was a higher frequency of intimate relationship problems in the past, a higher proportion of current unsafe living situation and a higher proportion of current personal problems.

**Table 3:** B-SAFER risk and vulnerability factors before and during the COVID-19 lockdown

**B-SAFER item**	**Before lockdown** (Jan 2016—February 2020)			**During lockdown** (March—December 2020)			
	Present	Total N of responses	%	Present	Total N of responses	%	*P*^*b*^
**Perpetrator risk factors**							
Violent acts, current	550	725	76	169	222	76	1.000
Violent acts, in the past	537	701	77	168	207	81	0.184
Violent threats or thoughts, current	294	697	42	91	190	48	0.563
Violent threats or thoughts, in the past	311	616	50	72	175	41	0.032
Escalation, current	315	673	47	113	197	57	0.010
Escalation, in the past	250	634	39	94	183	51	0.005
Violation of court orders, current	58	726	8	18	226	8	1.000
Violation of court orders, in the past	51	723	7	10	225	4	0.212
Violent attitudes, current	396	578	69	107	165	65	0.396
Violent attitudes, in the past	276	497	56	68	132	52	0.432
**Psychosocial adjustment**							
General criminality, current	215	724	30	80	226	35	0.118
General criminality, in the past	467	721	65	153	223	69	0.333
Intimate relationship problems, current	450	619	73	152	184	83	0.007
Intimate relationship problems, in the past^a^	356	570	62	140	175	80	0.000
Employment problems, current	261	602	43	90	182	49	0.149
Employment problems, in the past	205	549	37	74	171	43	0.178
Substance use problems, current	308	582	53	112	185	61	0.075
Substance use problems, in the past	328	584	56	120	187	64	0.610
Mental health problems, current	238	409	58	88	130	68	0.640
Mental health problems, in the past	212	381	56	85	130	65	0.064
**Victim vulnerability factors**							
Inconsistent behavior/ attitude, current	383	701	55	129	211	61	0.097
Extreme fear, current	251	642	39	91	196	46	0.690
Inadequate access to resources, current	188	615	31	69	191	36	0.156
Unsafe living situation, current^a^	384	649	59	161	220	73	0.000
Personal problems, current^a^	332	603	55	139	204	68	0.001

^a^Significant with α < 5% with Bonferroni correction

^b^Two-sided Fischer’s exact test

### Immigrants and differences in being perpetrator and victim before and during lockdown

During the overall study period of 2016–2020, 290 of the 974 perpetrators were immigrants which is a significant overrepresentation with SMR = 1.812, 95% CI = 1.796–1.828. Also, 156 of the 974 victims were immigrants, which was a small but significant overrepresentation with SMR = 1.013, 95% CI = 1.0003–1.026.

The overrepresentation of immigrants among the perpetrators was about the same before lockdown (January 2016-February 2020) as during lockdown (March-December 2020). 217 of the 745 perpetrators before lockdown, and 73 of the 229 perpetrators during lockdown were immigrants, representing an overrepresentation in both groups with SMR = 1.814, 95% CI = 1.792–1.836 before, and SMR = 1.807, 95% CI = 1.742–1.872 during lockdown.

The overrepresentation of immigrants among victims changed during lockdown. Before lockdown, 125 of the 745 victims were immigrants which was an overrepresentation with a SMR = 1.070, 95% CI = 1.052–1.087. On the contrary, during lockdown, only 31 of the 229 victims were immigrants, SMR = 0.835, 95% CI = 0.787-0.883 which was a significant underrepresentation of victims born outside of Norway.

### Age and gender of perpetrators and victims before and during lockdown

There were no differences in age before and during lockdown for neither perpetrators nor victims (Student’s t-test). The 745 perpetrators registered before lockdown had a mean age of 39.5, S.D. 12.0, range 18–83. The 229 perpetrators registered during lockdown had a mean age of 38.9, S.D. 12.5, range 19–85. The 745 victims registered before lockdown had a mean age of 36.4, S.D. 11.2, range 17–81. The 229 victims registered during lockdown had a mean age of 37.0, S.D. 12.5, range 18–76.

During lockdown there was a substantially higher proportion of female perpetrators. A total of 25 of the 229 (10.9%) perpetrators were women, as compared to the pre-lockdown period where 34 of the 745 (4.6%) perpetrators were women (Fischer’s exact test double-sided, *p* = 0.00124).

During lockdown, there was a substantially higher proportion of male victims. A total of 23 of the 229 (10.0%) victims were men, as compared to the pre-lockdown period where 34 of the 745 (4.6%) of the victims were men (Fischer’s exact test double-sided, *p* = 0.00344).

## Discussion

### Summary

In line with the growing literature assessing the link between the COVID-19 pandemic and IPV [5, 6, 8, 9], intimate partner violence in Norway, as reported to the police, increased substantially during lockdown compared to the pre-lockdown period. Similar results has been found in previous studies on intimate partner violence during the COVID-19 pandemic [9]. Furthermore, during lockdown the police assessed the cases as being at higher risk for imminent and severe violence. Notably, our findings indicated an over-representation among immigrant perpetrators, both before and during lockdown. While victims with immigrant backgrounds were over-represented in the pre-lockdown period, we found a significantly higher proportion of native Norwegian IPV victims during the lockdown period. Moreover, during lockdown, there were significantly more reports made to the police about intimate relationship problems, as well as female perpetrators and male victims.

### Comparisons with existing literature

As Norwegian health and social institutions locked down, and the availability of informal networks was reduced in 2020, the police service was one of a few institutions that remained open to the public. Our study found that during the lockdown period in Norway, police officers assessed an increased risk of violence in the near future among IPV victims, which may indicate a higher awareness among police officers about the potentially increased risk of family violence created by COVID-19 restrictions. Other studies have reported less access to medical services, as well as more severe injuries among IPV victims during COVID-19 lockdowns [26, 27]. Even though the finding in our study that the risk for severe violence was higher during lockdown did not reach statistical significance, it is still a relevant finding in a public health perspective [5, 6]. When the government initiated stay-at-home orders to avoid the spread of the COVID-19 virus, those individuals, already vulnerable to intimate partner violence, became trapped together with their perpetrator [7, 9]. As such, in the aftermath of this global crisis, health and social institutions may have to address the consequences of a pandemic of family violence.

According to the B-SAFER assessment tool, most cases in the study had a history of intimate partner violence, both pre-lockdown and during lockdown, which is in line with research showing that women who were already victims of intimate partner violence reported higher frequencies of violence than those who had not experienced violence before the lockdown [9]. In addition, the fact that in most cases the violence was recurrent indicates that those individuals who were already among the most vulnerable in society faced an even more difficult time during lockdown. Research on intimate partner violence has highlighted that violence within the family is associated with mental health problems, as well as drug use and economic difficulties [11, 12]. In this study, we found that most of the B-SAFER risk and vulnerability factors did not differ before and during the lockdown. However importantly, during lockdown significantly more victims were assessed as having personal problems and unsafe living conditions. In line with these findings, during lockdown the police in our study assessed a higher risk for IPV in the near future. The lockdown limited individuals’ ability to move outside of their home, which may have resulted in high psychological distress among the perpetrators and the victims. Hence, the victims were more vulnerable to incidents of violence during lockdown. This could perhaps also explain the increased proportion of female perpetrators during lockdown. Of significance, a cross-sectional study of self-reported violence in the early stages of the pandemic reported higher prevalence of male victims of intimate partner violence as compared to the general population [28]. Anxiety stemming from an insecure employment situation, staying home for long periods of time with children and partner, reduced possibilities to be with friends and wider family members, or colleagues at work, may all have impacted negatively on individuals’ ability to cope with intimate partner conflicts.

Another interesting finding was the higher proportion of native Norwegian victims of IPV during lockdown, as compared to immigrant victims. Intimate partner violence disproportionately affects marginalized groups like immigrants. Hence, one would expect an increased proportion of victims with an immigrant background during lockdown. Even though our study cannot draw any conclusions, one may speculate that our findings are associated with increased isolation among the immigrant population, less opportunity for immigrant victims to find safe havens and restricted access to health and social institutions during lockdown, which has been reported in previous studies [7, 9]. At the same time, native Norwegian victims, as opposed victims with immigrant background, may have more confidence that the police can help them, and were therefore more willing to contact the police.

### Strengths and limitations

A strength of this study was that it included all cases reported to the police in a defined geographical region. Moreover, the study period of the lockdown was 10 months, which enabled the researchers to observe consequences over time.

On the other hand, our study only included information about the cases reported to the police. Consequently, since we do not know if the propensity to report to the police has changed during lockdown, we cannot say anything about changes in actual numbers of intimate partner violence in the population during lockdown.

## Conclusions

In this study we found a substantial increase in the number and severity of reported cases of intimate partner violence during lockdown in Norway. The results indicate a need to create a wide range of options for victims to leave their perpetrator, particularly during times of crisis, and with special focus on assisting the most vulnerable victims in society.

## Data Availability

All data generated or analysed during this study were included in this published article.

## Abbreviations

B-SAFER: The Brief Spousal Assault Form for the Evaluation of Risk
CI: Confidence Interval
DPIA: Data Protection Impact Assessment
GDPR: General Data Protection Regulation
IPV: Intimate Partner Violence
SD: Standard deviation
SMR: Standard Morbidity Rate
